# The Conductivity and Dielectric Properties of Neobium Substituted Sr-Hexaferrites

**DOI:** 10.3390/nano9081168

**Published:** 2019-08-15

**Authors:** B. Unal, M. Almessiere, Y. Slimani, A. Baykal, A. V. Trukhanov, I. Ercan

**Affiliations:** 1 Institute of Forensic Sciences and Legal Medicine, Istanbul University-Cerrahpaşa Buyukcekmece Campus Alkent 2000, Buyukcekmece-Istanbul 34500, Turkey; 2 Department of Biophysics, Institute for Research and Medical Consultations (IRMC), Imam Abdulrahman Bin Faisal University, Dammam P.O. Box 34221, Saudi Arabia; 3 Depaertement of Physics, College of Science, Imam Abdulrahman Bin Faisal University, Dammam P.O. Box 34221, Saudi Arabia; 4 Department of Nanomedicine, Institute for Research and Medical Consultations (IRMC), Imam Abdulrahman Bin Faisal University, Dammam P.O. Box 34221, Saudi Arabia; 5 National University of Science and Technology MISiS, 119049 Moscow, Russia; 6 SSPA “Scientific and Practical Materials Research Centre of the NAS of Belarus”, 220072 Minsk, Belarus

**Keywords:** SrFe_12_O_19_, rare earth substitution, impedance spectroscopy, dielectric, conductivity

## Abstract

The Nb3+ ion substituted Sr hexaferrites (SrNb_x_Fe_12−x_O19 (x = 0.00–0.08) hexaferrites (HFs)) were fabricated via a citrate-assisted sol-gel approach. X-ray powder diffractometer analysis affirmed the pureness of all products. The crystallite sizes of the products which were estimated from Scherrer equation were in the 36–40 nm range. The chemical component of the samples was proved by Energy-dispersive X-ray spectroscopy (EDX) and Elemental mapping. The hexagonal morphology of all products was confirmed by Field Emission Scanning Electron Microscopy (FE-SEM). The electrical conduction mechanisms and dielectric properties of a variety of Nb3+ions-substituted SrNb_x_Fe_12−x_O19 HFs were investigated by a complex impedance system. Dielectric parameters such as conductivity, dielectric constant, dielectric loss, dielectric tangent loss and complex modulus, were studied at temperatures up to 120 °C in a frequency range varying from 1.0 Hz to 3.0 MHz for several Nb ratios. The frequency dependence of the conductivity was found to comply with the power law with diverse exponents at all frequencies studied here. Subsequently, incremental tendencies in dc conductivity with temperature indicate that the substituted Sr-HFs leads to a semiconductor-semimetal like behavior. This could be attributable to a feature of conduction mechanism which is based on the tunneling processes. Additionally, the dielectric dispersion pattern was also explained by Maxwell–Wagner polarization in accordance with the Koop’s phenomenological theory.

## 1. Introduction

M-type hexagonal ferrites are used in many special applications especially on communications, sensors, electronics and radar absorbing materials [1]. Due to their low cost production, high Curie temperature, large magneto-crystalline anisotropy, superior coercivity and considerable saturation magnetization as well as amazing corrosion resistance and chemical stability [2], these materials are excellent hard ferrite candidates for using as high frequency [3], microwave components [4], high-density magnetic memory media [5] and magneto optical devices [6].

Many researchers focused on enhancing electo-magnetic properties of SrFe_12_O_19_ through different approaches such as conventional solid oxide reaction [7], chemical co-precipitation [8,9,10], hydrothermal method [11], sol-gel [12], combustion method [13,14], etc. The main purpose is to change its magneto-crystalline anisotropy from uniaxial to either planar/cone basal plane crystal structure resulted in free rotation of magnetization in the plane/cone in order to obtain a perfect magnetic performance in the GHz range and a higher resonance frequency. Moreover, dielectric losses of ferrites are necessary to be minimized for high frequency applications since the damping of the vibration of electrical dipoles occurs at higher frequency, therefore; eliminating the permittivity provides full penetration of the electric field. For this purpose, M-ferrites or specifically Stronium hexaferrites are substituted with different cations and rear earth ions such as Ba-Mn, Zn [15], Ni-Zr [16], La-Co [17], Nb-Zn [18], Nb [19], and Tb [20].

In this work, Nb substituted strontium hexaferrites were produced via a citrate-assisted sol-gel approach and their structural, morphological characterizations, conductivity and dielectric properties were investigated.

## 2. Experimental Procedure

### Synthesis and Instrumentations

Sr((NO3)2, Fe(NO3)3•6H2O, NbCl3 and C6H8O7 were obtained from Merck (City, US State abbrev. if applicable, Country) and used to fabricate SrNb_x_Fe_12−x_O19 (0.00–0.08) HFs by a citrate-assisted sol-gel combustion approach. The metal salts were dissolved in Deionized water with stirring for at 90 °C for 60 min. The solution pH was arranged at 7 by NH3 with continuous stirring then the temperature was raised to 160 °C for 40 min to evaporate water in order to obtain a gel which was then heated to 350 °C and burnt to become a black powder. The final solid powder was calcinated at 1050 °C for 5 h. For the structural investigation, X-ray diffraction (XRD) (Rigaku, Benchtop Miniflex (Cu Kα), Tokyo, Japan) was used. The FE-SEM (FEI Titan S/TEM, Hillsboro, OR, USA) with EDX was used for morphological analysis. For EDX analysis, a different beam setting has been applied, 20 kV of acceleration voltage and 0.8 nA of probe current, to have a good signal-to-noise ratio, and collect the X-ray signals of heavier elements. The dielectric measurements of samples were done via a Novocontrol Alpha-N high-resolution dielectric-impedance analyzer (Montabaur, Novocontrol Technologies GmbH & Co. KG, Germany).

## 3. Results and Discussion

### 3.1. Structure

**Figure 1:**
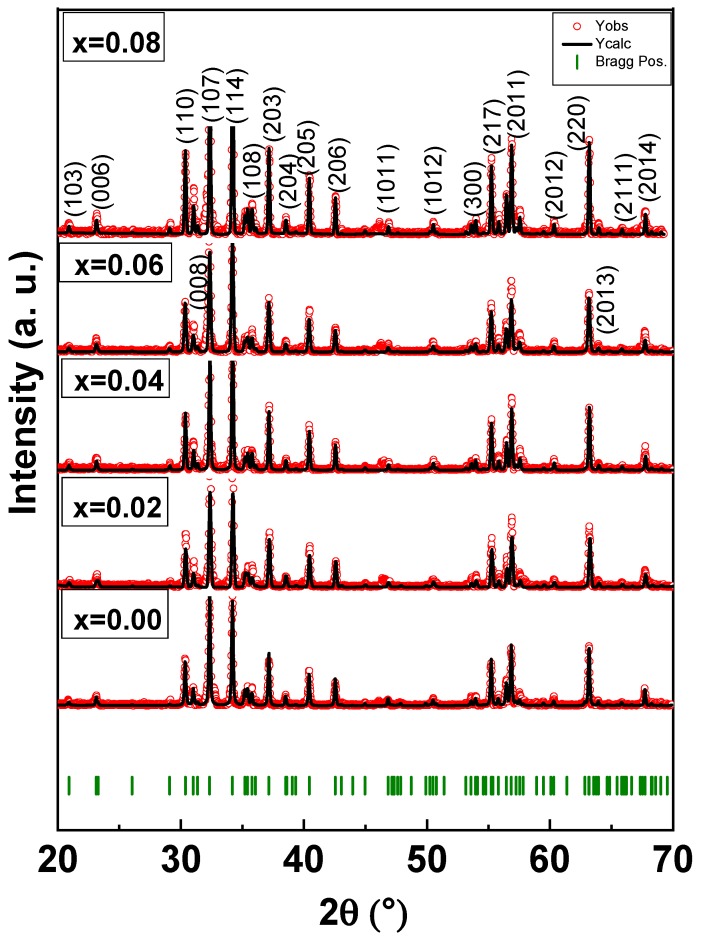
XRD powder patterns of SrNb_x_Fe_12−x_O_19_ (0.00 ≤ x ≤ 0.08) HFs.

The XRD pattern of SrNb_x_Fe_12−x_O_19_ (0.00–0.08) HFs was recorded in Figure 1. The spectrum showed the indexed peaks of Sr- HFs (according to JCPDS Card 00-043-0002) without the presence of any impurities. The structural parameters were calculated by using Fullproof (version, Manufacturer, City, US State abbrev. if applicable, Country) and presented in Table 1. The lattice constant “a” is almost steady, where “c” increased with the raising Nb content as a result of the higher ionic radius of Nb^3+^ (0.72 Å) than Fe^3+^ (0.64 Å). The crystallite size is in the range of 35 to 40 nm [21,22].

### 3.2. Morphological Analysis

**Figure 2:**
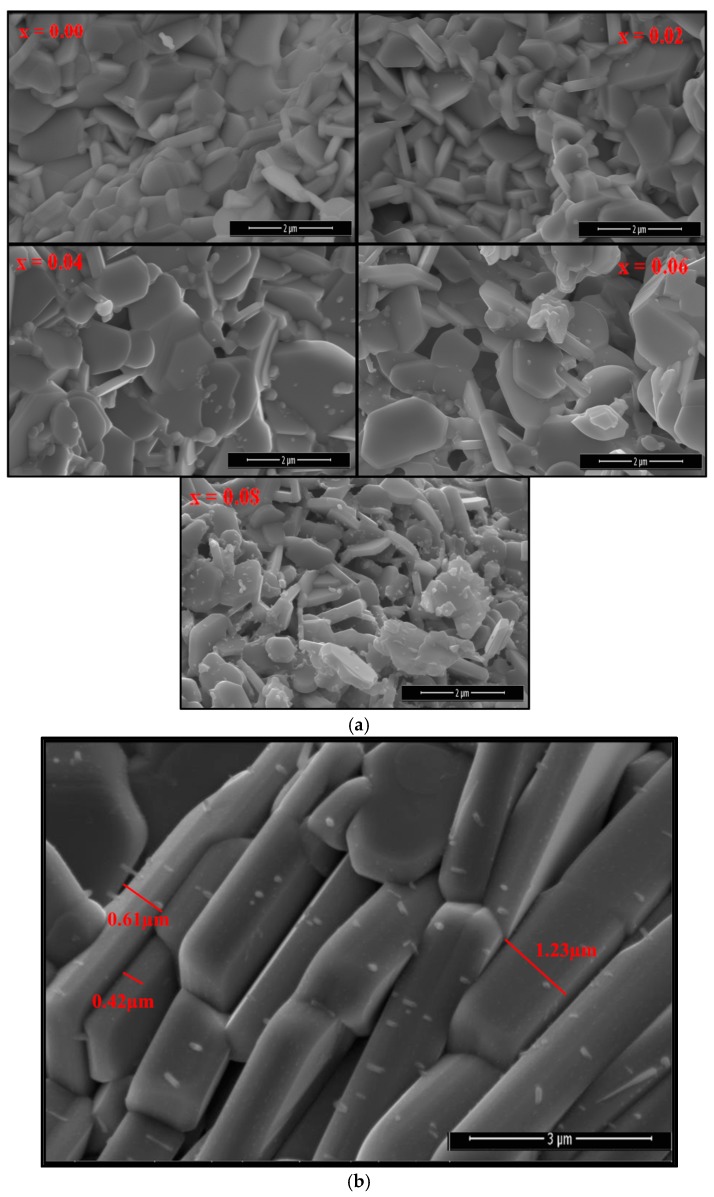
(**a**) FE-SEM micrographs of SrNb_x_Fe_12−x_O19 (0.00 ≤ x ≤ 0.08) HFs; (**b**) cross section of SrNb_x_Fe_12−x_O19 (0.08) HFs.

**Figure 3:**
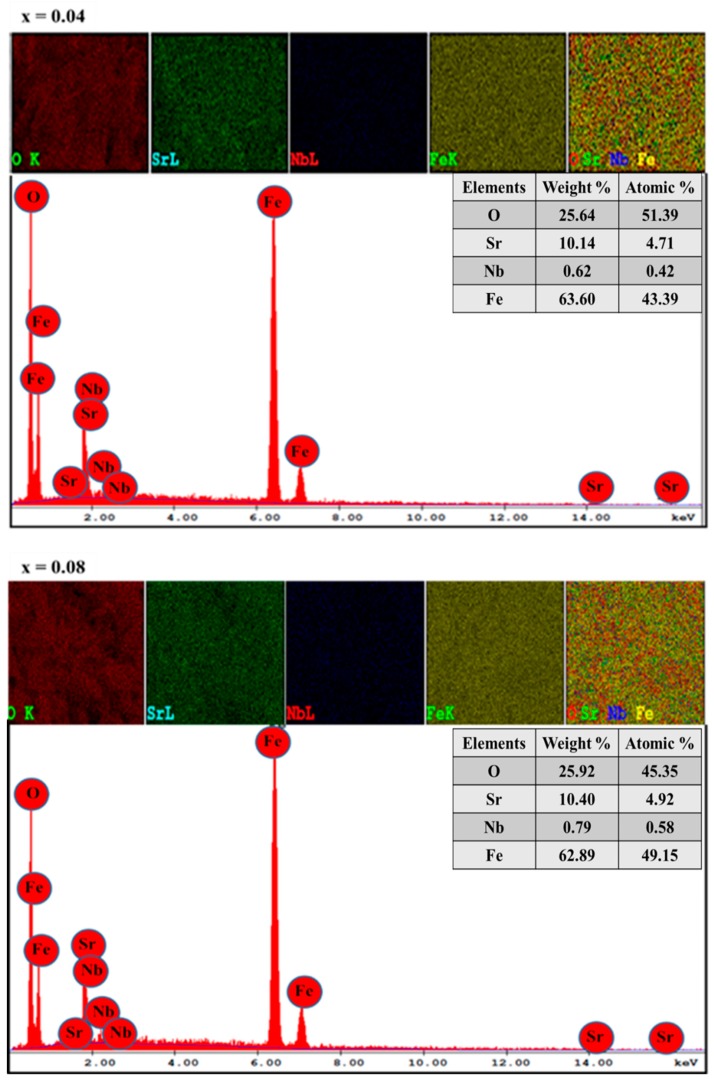
EDX patterns and elemental mapping results of SrNb_0.04_Fe_11.96_O_19_ and SrNb_0.08_Fe_11.92_O_19_ (0.00 ≤ x ≤ 0.08) HFs.

The FE-SEM micrograph of SrNb_x_Fe_12−x_O19 (0.00–0.08) HFs is seen in Figure 2. It is noticeable that the particles have aggregations of hexagonal disks produced from a large group of particles as displayed in Figure 2a. Figure 2b showed the magnification cross section image of SrNb_x_Fe_12−x_O19 (0.08) HFs; it is found that the thickness of hexagonal disks is in the range of 0.6 to 1.23 µm. In addition, it is found that the average particle size does not show a significant change while increasing the substitution content. EDX patterns and elemental mapping results of SrNb_x_Fe_12−x_O19 (x = 0.04 and 0.08) HFs are recorded in Figure 3, specifying the stoichiometric ratios of elements that make up the substituted Sr-HFs.

### 3.3. Impedance Spectroscopy

For a substitutional variation of many hexaferrites, it is evident to note that the frequency-dependency over conductivity is a good approach to study their conduction mechanism. The conduction mechanism of most ferrites can be assigned to two parts; the first part is the band correlated conduction as a DC conductivity [23,24], and the second part is the hopping related conduction mechanism as an AC conductivity among the same ions in hexaferrites occurring in more than a single valence state. This trend will be expressed as an exponent power law dependency, which will be discussed later.

The AC/DC conductivity, dielectric constant and dielectric loss including tangent loss (tanδ) as a dissipation factor are all analyzed as functions of frequency, temperature, and for substitutional composition and ratios, utilizing an impedance analyser within the frequencies up to 3 MHz and temperatures between 20 °C and 120 °C, and substitution ratios in the range of x = 0.00–0.08.

#### 3.3.1. AC Conductivity

**Figure 4:**
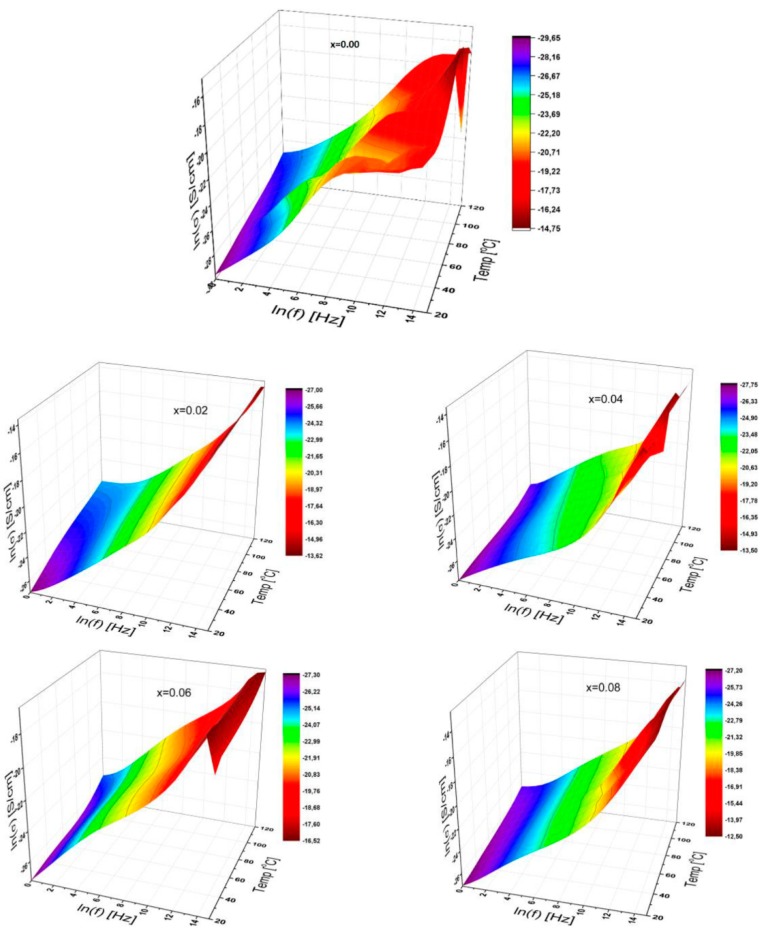
3D characteristic plots of *AC* conductivity of SrNb_x_Fe_12−x_O_19_ (0.00 ≤ x ≤ 0.08) HFs for various Nb doping ratios as a function of frequency up to 3 MHz, and for temperatures up to 120 °C.

The 3D characteristic plots of conductivity of SrNb_x_Fe_12−x_O_19_ HFs for substitution ratios x = 0.00; 0.02; 0.04; 0.06; 0.08 are displayed in Figure 4 as functions of frequency ranging up to 3 MHz, and for temperatures between 20 °C and 120 °C in the interval of 10 °C. This frequency-dependency over conductivity for all samples is acquired from the following equation [25,26]:

(1) $$\sigma^{\prime }\left(\omega ,T;x\;\right)=\sigma_{ac}\left(\omega ,T;x\right)=\varepsilon \prime \prime \left(\omega ,T;x\right)\omega \varepsilon_{0},$$

where *σ’(ω, T; x)* is the real part of complex conductivity and equals to ac conductivity of *σ_ac_(ω, T; x)*, ω is the angular frequency of *ac* signal applied across the coupled electrodes, ε″*(ω, T; x)* is the dielectric loss, and *T* is the temperature in *K*, *ε*_0_ is the vacuum dielectric permittivity of 8.852 × 10^−12^ *F/m*) and finally x is the ratio of Nb ion substitution in Sr-hexaferrite.

Thus, it is well known that any correlations of conductivity with both temperature and concentration as a function of frequency give us important information to understand the conduction mechanism. It is evident to comprehend that the *AC* conductivity for all SrNb_x_Fe_12−x_O_19_ (0.0 ≤ x ≤ 0.08) HFs increases with a frequency ranging up to 3 MHz, while fewer effects are recorded via temperature variation between 20 °C and 120 °C. However, at higher frequencies, the conductivity fluctuates slightly except for x = 0.02 and 0.08, possibly attributable to compositional distribution caused by grain-grain boundaries. The 3D conductivity curve contains a variety of plateaus in the medium/higher frequency ranges for unsubstituted HFs. It should be noted that these plateaus occur by changing the temperature as well.

Furthermore, when a small amount of the *Nb*^3+^ ions concentration in Sr-HFs is increased, the *DC* conductivity rises to a value of 0.895 pS/cm for x = 0.02; 1.454 pS/cm for x = 0.06 and finally 1.615 pS/cm for x = 0.08, attributable to the conduction mechanism of ferric and ferrous ions. Additionally, at medium and higher frequencies, the level of AC conductivity fluctuates with the variation of *Nb^3+^* ion concentrations while obeying the exponent power law of the signal frequency.

The level of *Nb*^3^ ions concentration causes the conductivity to become saturated at high frequencies, except for a curve labeled as x = 0.02 and 0.08. Thus, the functionality of AC conductivity for x = 0.04 is duplicated for the two exponent dependencies of power law although unsubstituted Sr-HFs are observed to be highly deep fluctuated at medium and high frequencies.

#### 3.3.2. DC Conductivity

The *DC* electrical conductivities (σ_DC_) of SrNb_x_Fe_12−x_O_19_ HFs were derived from the well-developed plateau region in 3D graphs of natural *logσ_ac_* versus *logf* by linear fittings at a frequency of 1 Hz. The DC conductivities versus reciprocal temperature up to 120 °C are calculated for each ferrite (0.00 ≤ x ≤ 0.08). Accordingly, *DC* conductivity can be expressed as a well-known Arrhenius plot for the entire substitutional Nb ion ratios. From Figure 4, the linearity of each curve changes slightly with the substitutional ratio. It has been indicated that the conductivity of Nb ion-substituted Sr-HFs is thermally activated depending upon a certain temperature ranges, and for both cases it can be expressed using the following Arrhenius relation as mentioned earlier:

(2) $$\sigma_{dc}\left(T\right)=\sigma \left(0\right)\exp\left[-\frac{E_{a}}{k_{B}T}\right],$$

where *σ_DC_* stands for DC conductivity, *σ*(0) is the pre-exponential term, *E_a_* is the activation energy, *k_B_* is the Boltzmann constant (8.617 × 10^−5^ eV.K^−1^) and *T* is the temperature in *K*. The activation energy may reveal some fluctuating trends by changing the Nb^3+^ ions substitution ratios. This can be explained as more energy being required for active charge carriers to hop from one cationic site to another by increasing the number of both *doping* ions. Thus, resulting tendencies may cause a drop in the *DC* conductivity to some extent. Subsequently, incremental tendencies in conductivity with the temperature indicate that the substituted Sr-HFs show a semiconductor-semimetal like behavior.

#### 3.3.3. Dielectric Constant

**Figure 5:**
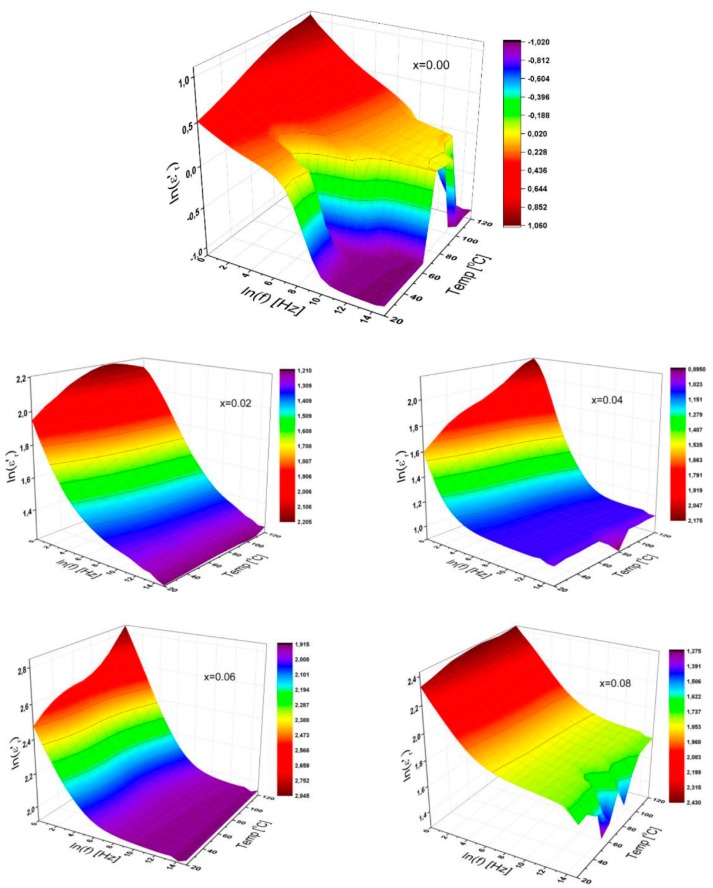
3D characteristic plots of dielectric constant of SrNb_x_Fe_12−x_O_19_ (0.00 ≤ x ≤ 0.08) HFs for a variety of substitutional Nb ions ratio as functions of frequency up to 3 MHz, and for temperatures up to 120 °C.

The frequency- and temperature-dependency of the complex permittivity of substituted Sr HFs in a frequency ranging up to 3 MHz and for temperatures up to 120 °C were represented in Figure 5 for all substitutions of 0.00 ≤ x ≤ 0.08.

The 3D characteristic representation depicts that the dielectric constant for all the SrNb_x_Fe_12−x_O_19_ (0.00 ≤ x ≤ 0.08) HFs decreases sharply with the increase of frequency. However, the rate of reduction is almost different for all HFs depending on temperature and substitution ratio. For pure Sr-HF, a sharp valley formation exists at a temperature of about 60 °C for medium frequencies, and it is then expanded along both the higher frequency and lower temperature sides while another sharp drop is observed at higher frequency over 1 MHz and higher temperature above 110 °C.

It can be interpreted that the characteristic variation of relative dielectric constant with an externally applied frequency is attributable to the occurrence of the electrode interface polarization processes, which is dominated at lower frequencies. The profile of 3D curves remains quite different for a variety of substitutional ratios, and its natural logarithmic value for each ratio varies between 0–1 for x = 0.00; 0.20–0.75 for x = 0.02; 0.89–2.20 for x = 0.04; 1.90–2.85 for x = 0.06 and 1.25–2.45 for x = 0.08. Some similar tendencies can be observed in the 3D curves especially for x = 0.00 and 0.08 even at higher frequencies.

It is well known that the dielectric constant can be associated with how fast the degree of polarization in a material is tracked so that it can monitor the oscillations of a variable electric field. Thus, the orientation polarization can be reduced by increasing the frequency because the alignment of the dipole moments requires a longer time interval than the electronic and ionic polarizations, leading to a reduction in the dielectric constant.

When related to temperature, the dielectric constant generally increases with the temperature due to molecular orientation and regulation [27]. Thus, the dielectric constant of SrNb_x_Fe_12−x_O_19_ (0.00 ≤ x ≤ 0.08) HFs increases with the increase of temperature at lower frequency because of the enhancement of grain–grain boundaries caused by the dopant ions by varying a substitutional Nb^3+^ ions ratios in Sr-HFs.

It should be emphasized that any increase in thermal energy can lead to a significant increase in the mobility of the charge carriers and the measured effective permittivity contains a comprehensive dependence on both the microstructure and the permittivity of any nanostructural compositions [28], which could be clarified by the Clausius–Mosotti equation [29]. This obtained result may interpret the relationship between dielectric constant and polarizability coefficient of its dipole for any substance. Hence, any reduction in polarization conditions under the temperature drop may result in a significant reduction in dielectric permittivity of the Sr-HFs as shown in Figure 5.

#### 3.3.4. Dielectric Loss

**Figure 6:**
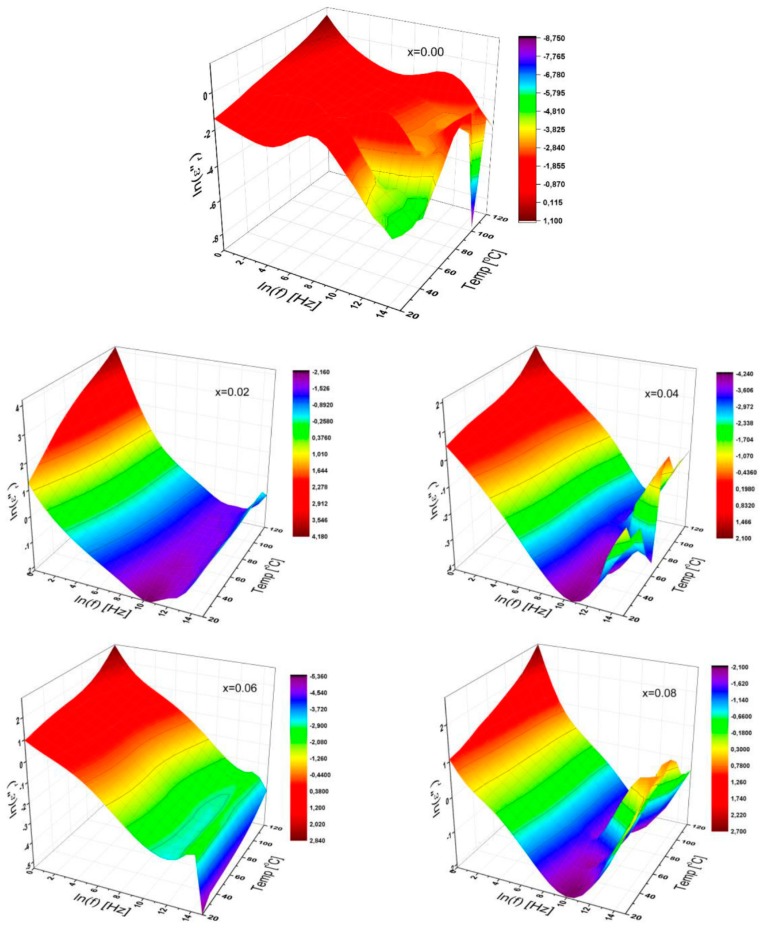
3D characteristic plots of dielectric loss of SrNb_x_Fe_12−x_O_19_ (0.00 ≤ x ≤ 0.08) HFs for a variety of substitutional Nb ions ratio as functions of frequency up to 3 MHz, and for temperatures up to 120 °C.

The 3D characteristic plots of dielectric loss of SrNb_x_Fe_12−x_O_19_ (0.00 ≤ x ≤ 0.08) HFs as functions of frequencies up to 3 MHz are depicted in Figure 6 for temperatures up to 120 °C from 20 °C

Generally, both the frequency and temperature dependence of dielectric loss for a variety of substituted Sr-HFs demonstrates a variety of tentative decrease with frequency at low and medium frequency regime. Thus, this is more significant at higher temperatures for all; however, in contrast, all of the curves lead us to a variety of tendencies at higher frequencies being subject to the substitutional *Nb*^3+^ ions’ ratios. This linearity part at the lower frequencies of the natural log–log graph corresponds to the *DC* conductance (σ_DC_) with the following equation:

*ε*”_*DC*_ (*ω*, *T*; *x*) = *ωC_o_σ_DC_*(*T*; *x*). (3)

Here, *ω* is the angular frequency; *C_o_* is the vacuum capacitance; and it is noted that, for the parameter calculations, the separation between double electrode plates equals the sample thickness. It was found that the conduction mechanism is linked to the temperature effect and the redistribution process resulted from the structural diffusion among the elemental configurations in ferrites. It is evident that the capacitive feedback of the particles reveals a high temperature-dependent consistency instead of the reorganization nature in the substituted Sr-HFs. Thus, the decline in dielectric loss of SrNb_x_Fe_12−x_O_19_ HFs eventually reached a minimum at a certain frequency relevant to the degree of substitution ratio, and then the loss is changed over the higher frequency side relying upon the substitutional ratio of Nb ions in Sr-HFs, while, for unsubstituted Sr-HFs fluctuating at high frequencies, it rises up with the elevated temperatures.

Furthermore, the dielectric loss of the SrNb_x_Fe_12−x_O_19_ HFs decreases with the frequency increase. Then, the dielectric properties of any heterogeneous structure can also be elucidated using the Koop’s theory [29] on the basis of the Maxwell–Wagner model. The basis of the dielectric structure is thought to be composed of conductive ferrite grains possessing a very good construction that is electrically-isolated by fine-grained boundary layers. These grain boundaries appear as a result of the superficial reduction and/or oxidation of the micro-crystallites [30]. It has been found that grain boundaries are efficient in low conductivity at low frequencies while hexaferrite grains are more effective at high frequencies in higher conductivity [30]. Such tendencies pointed out that there is a reduction in both dielectric constant and loss with the incremental frequencies.

#### 3.3.5. Dielectric Modulus

**Figure 7:**
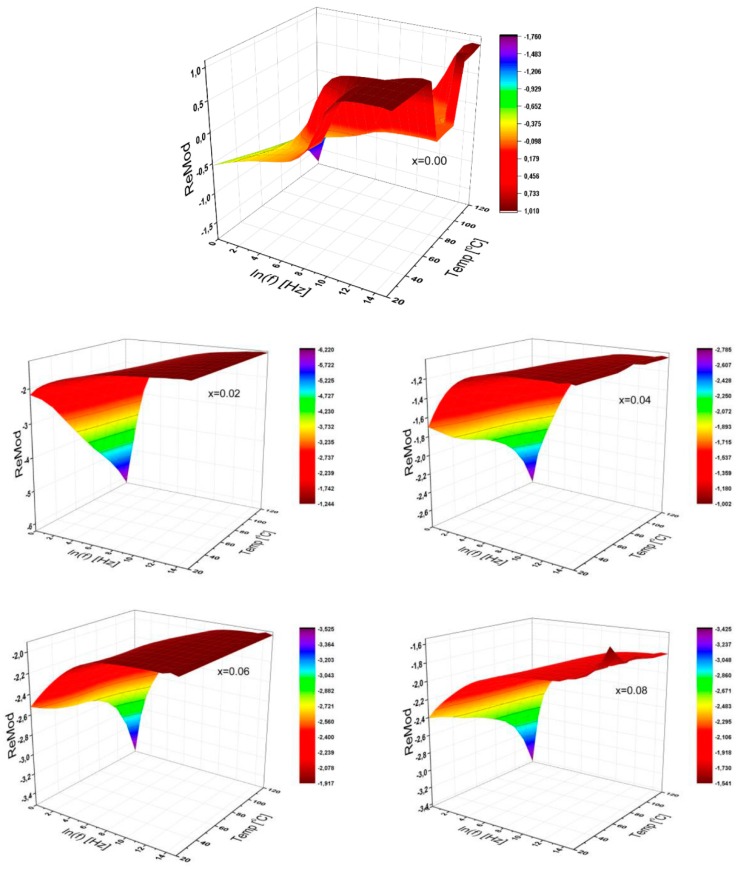
3D characteristic plots of real modulus of SrNb_x_Fe_12−x_O_19_ (0.00 ≤ x ≤ 0.08) HFs for a variety of substitutional Nb ions ratio as functions of frequency up to 3 MHz, and for temperatures up to 120 °C.

The 3D representations of the real part of dielectric modulus of SrNb_x_Fe_12−x_O_19_ (0.00 ≤ x ≤ 0.08) HFs were presented in Figure 7 as functions of frequency ranging up to 3.0 MHz for temperatures valued from 20 °C to 120 °C. Each curve for all the substitution ratios is leading to a similar tendency for the parametric variation of both temperature and frequency while that of Sr-HFs representations fluctuate with the elevated temperatures and by increasing frequency. The magnitude of the real modulus is varied randomly with the ionic ratios as shown in Figure 7.

In general, it has been found that the real part of dielectric module for all-doped samples is increased with the elevated temperature at low frequencies following various slopes and remains almost constant at high frequencies, while, for Sr-HFs, it fluctuates for both regions.

**Figure 8:**
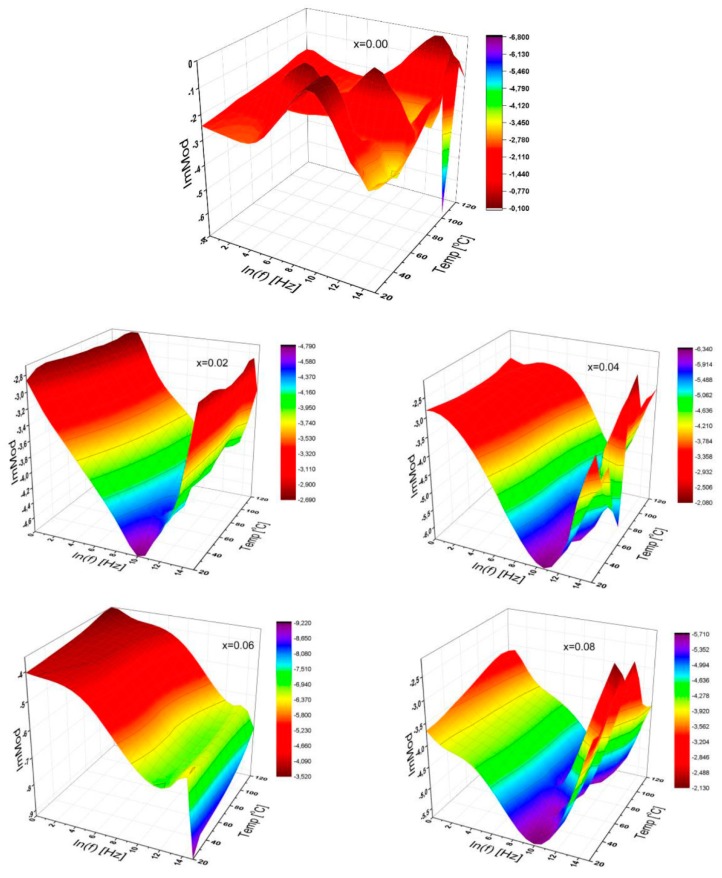
3D characteristic plots of imaginary modulus of SrNb_x_Fe_12−x_O_19_ (0.00 ≤ x ≤ 0.08) HFs for a variety of substitutional Nb ions ratio as functions of frequency up to 3 MHz, and for temperatures up to 120 °C.

The 3D representation for the imaginary modulus of SrNb_x_Fe_12−x_O_19_ (0.00 ≤ x ≤ 0.08) HFs was depicted in Figure 8 as functions of frequency ranging up to 3 MHz, and for temperatures between 20 °C and 120 °C in the interval of 10 °C.

Only the Sr-hexaferrite itself exhibits a notably higher fluctuation for dielectric modulus, while the Nb substituted Sr-HFs show a quite different variation to us as shown in the last five graphs in Figure 8. Especially for the substitutional ratios of x = 0.02; 0.04 and 0.08, the imaginary modulus illustrates V-shape tendencies and slightly shifts its minimum value from the frequency of 22 kHz to 36 kHz, while there seems to be a completely different manner for the curves with the substitutional ratios of 0.06. These results indicate that each substitutional Nb ion level presents a significant influence on the dielectric module.

#### 3.3.6. Dissipation Factor

**Figure 9:**
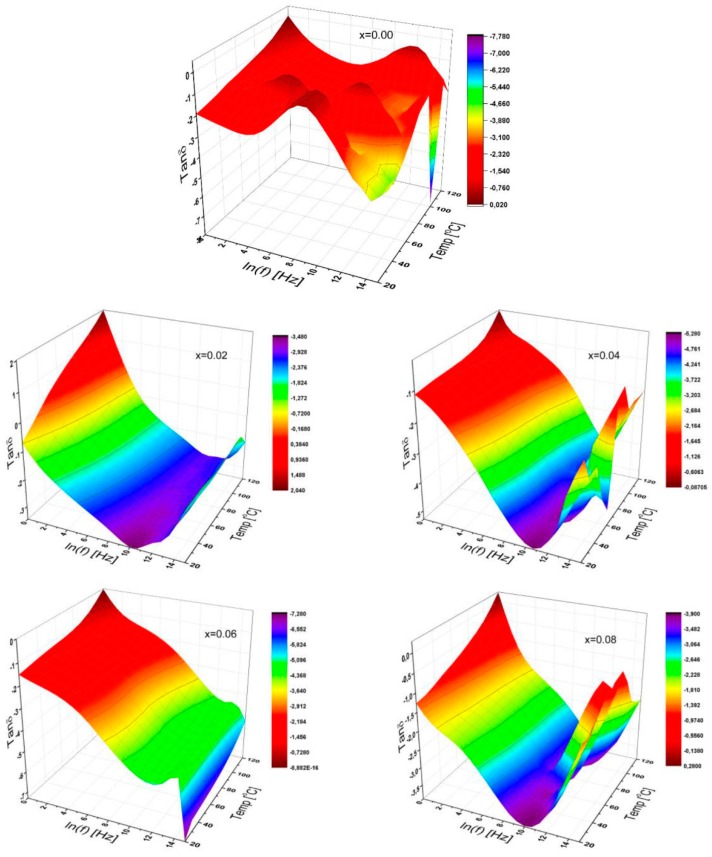
3D characteristic plots of dielectric tangent loss of SrNb_x_Fe_12−x_O_19_ (0.00 ≤ x ≤ 0.08) HFs for a variety of substitutional Nb ions ratio as functions of frequency up to 3 MHz, and for temperatures up to 120 °C.

The dielectric tangent loss is also known as dissipation factor, which is the ratio of dielectric loss to the dielectric constant, of the substituted Sr-HFs as a function of frequency at different temperatures for a variable substitutional ratio, which is illustrated in Figure 9.

It can be clearly seen from Figure 9 that the dissipation factor, especially at low frequencies, appears to increase for each of the variably substituted Sr-HFs for all temperatures. Furthermore, the dissipation factor shows reductions with increasing frequency ready for a specific value depending on Nb ion ratios, and then grows up to a certain level depending on the Nb ion ratios again and fluctuates at higher frequencies, especially for certain substitutional ratios of x = 0.04 and 0.08. The absolute value of the dissipation factors for the substitutional ratios of x = 0.01 and 0.06 increases with a variety of the manners depending on the effect of the Nb ion substitution ratios over dielectric properties. At very high frequencies, there may be significant differences in the dielectric tangent loss. For non-substituted Sr-HFs, the distribution issue at any temperatures above 90 °C can provide us with very different trends, so both conductivity and permittivity give us more information about the origin of conduction mechanisms [31].

Moreover, for some Nb ion substitutional ratios of the sample, the dissipation factor reaches a maximum value, which is shifted to the elevated frequency side as temperature increases. In addition, the dissipation factor for samples with a certain substitution ratio such as 0.02; 0.04; and 0.08 reaches a maximum value by shifting to higher frequencies as the temperature elevates. V-shape curves for the rest become expanded when the substitution ratio is 0.01 and 0.06. Thus, this can be attributed to any influential dipoles oriented by the alternative electric field as discussed in the literature [32,33,34].

## 4. Conclusions

The SrNb_x_Fe_12−x_O_19_ (0.00–0.08) HFs have been synthesized by a citrate-assisted sol-gel route. The microstructure, morphology and composition of products were confirmed through XRD and FE-SEM, EDX and elemental mapping of the stoichiometric of elements in SrNb_x_Fe_12−x_O_19_ (0.00–0.08) HF compositions. The electrical characterization of the Sr-NHFs revealed that the AC conductivity complies with a power base law having an exponent, n, for the entire Nb ion ratios. The Nb ion substitution can result in a better stability over the electrical bonding formed amongst the substitutional Nb^3+^ ions and the host Fe^2+^ ions in Sr-NHFs. Likewise, substitutional Nb ion ratios supplied us with a better optimization and tunability in the conductivity, dielectric values of both constant and loss in Sr-hexaferrites.

## Figures and Tables

**Table 1:** Structural parameters of SrNb_x_Fe_12−x_O_19_ (0.00 ≤ x ≤ 0.08) HFs.

x	a = b (Å)	c (Å)	V(Å^3^)	D_XRD_ (nm)
**0.00**	5.882	23.0427	690.6826	36.0
**0.02**	5.886	22.9580	688.0110	36.6
**0.04**	5.880	23.0230	688.2945	40.8
**0.06**	5.882	23.0236	689.8543	35.3
**0.08**	5.880	23.0278	689.8543	37.3
